# Bilosomes as Nanocarriers for the Drug and Vaccine Delivery against Gastrointestinal Infections: Opportunities and Challenges

**DOI:** 10.3390/jfb14090453

**Published:** 2023-09-01

**Authors:** Elham Zarenezhad, Mahrokh Marzi, Hussein T. Abdulabbas, Saade Abdalkareem Jasim, Seyed Amin Kouhpayeh, Silvia Barbaresi, Shiva Ahmadi, Abdolmajid Ghasemian

**Affiliations:** 1 Noncommunicable Diseases Research Center, Fasa University of Medical Sciences, Fasa P.O. Box 7461686688, Iran; el.zarenezhad@gmail.com (E.Z.); m.mahrokh89@gmail.com (M.M.); drshivahmadi@gmail.com (S.A.); 2 Department of Medical Microbiology, Medical College, Al Muthanna University, Al Muthanna P.O. Box 07835544777, Iraq; hussbiooo@gmail.com; 3 College of Applied Science, University of Fallujah, Fallujah, Anbar 31002, Iraq; saadee198814@gmail.com; 4 Department of Pharmacology, Faculty of Medicine, Fasa University of Medical Sciences, Fasa P.O. Box 7461686688, Iran; kouhpayeha@gmail.com; 5 Department of Movement and Sports Sciences, Ghent University, 9000 Ghent, Belgium; silvia.barbaresi@ugent.be

**Keywords:** bilosomes, gastrointestinal infections, drug delivery, vaccines

## Abstract

The gastrointestinal tract (GIT) environment has an intricate and complex nature, limiting drugs’ stability, oral bioavailability, and adsorption. Additionally, due to the drugs’ toxicity and side effects, renders are continuously seeking novel delivery systems. Lipid-based drug delivery vesicles have shown various loading capacities and high stability levels within the GIT. Indeed, most vesicular platforms fail to efficiently deliver drugs toward this route. Notably, the stability of vesicular constructs is different based on the different ingredients added. A low GIT stability of liposomes and niosomes and a low loading capacity of exosomes in drug delivery have been described in the literature. Bilosomes are nonionic, amphiphilic, flexible surfactant vehicles that contain bile salts for the improvement of drug and vaccine delivery. The bilosomes’ stability and plasticity in the GIT facilitate the efficient carriage of drugs (such as antimicrobial, antiparasitic, and antifungal drugs), vaccines, and bioactive compounds to treat infectious agents. Considering the intricate and harsh nature of the GIT, bilosomal formulations of oral substances have a remarkably enhanced delivery efficiency, overcoming these conditions. This review aimed to evaluate the potential of bilosomes as drug delivery platforms for antimicrobial, antiviral, antifungal, and antiparasitic GIT-associated drugs and vaccines.

## 1. Introduction 

**Figure 1:**
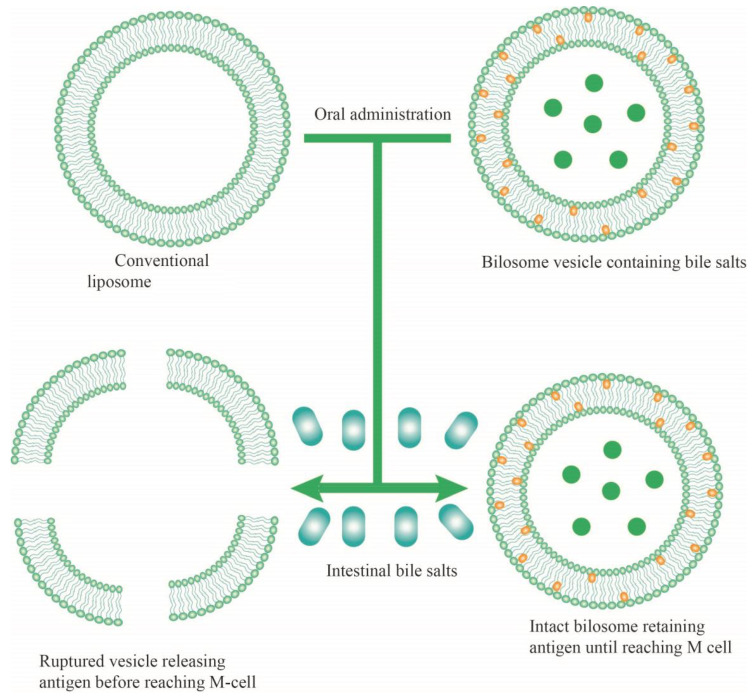
The stability of bilosomes compared to liposomes upon exposure to GIT bile salts. Bilosomes are composed of lipid bilayer and bile salts. Niosomes also undergo degradation similar to liposomes.

**Figure 2:**
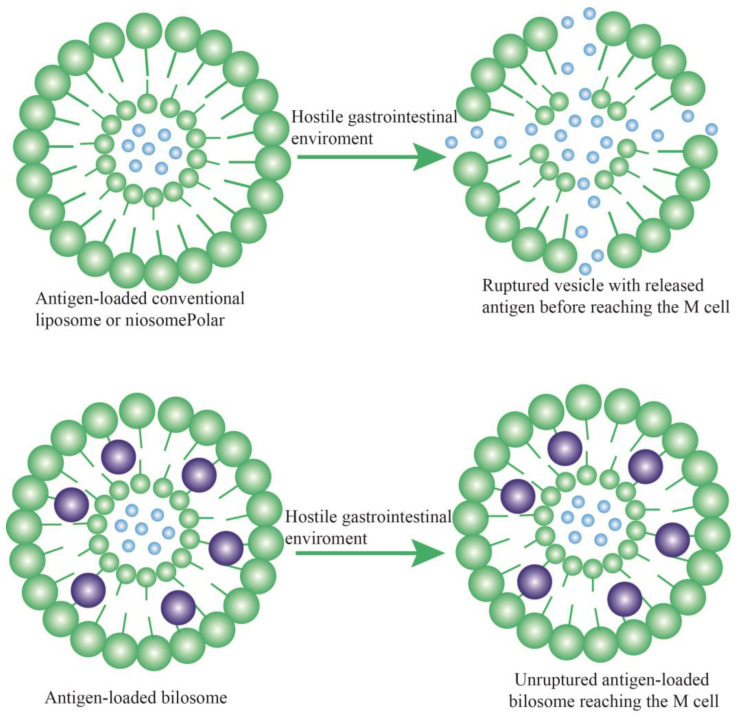
The protective effects of bile salts within a vesicular structure (bilosome) from the harsh GIT environment [16].

The gastrointestinal tract (GIT) infections include numerous bacterial, viral, parasitic, and fungal agents [1,2]. The intricate nature and harsh conditions of the GIT limit the stability and bioavailability of oral drugs or vaccines. Even nanocarriers possibly undergo degradation within the GIT [3,4,5,6]. On the other hand, the increasing problem of drug resistance in healthcare and in the global community facing microbial infections has raised concerns regarding the efficacy of last-line drugs, and there is an urgent need to develop novel agents [7,8]. In addition, side effects and costs associated with synthetic antimicrobials have increased, leading researchers in the field to seek novel and efficient delivery approaches. Lipid-based carriers such as niosomes, liposomes, exosomes, and bilosomes are among the most common drug or vaccine delivery systems currently used in humans, each with their own promises and/or drawbacks [9,10]. The delivery of various materials throughout the GIT requires vesicular or drug stability, sufficient absorption and bioavailability, and efficient release and penetration into the intestinal epithelial cells. Bilosomes are nonionic, amphiphilic, flexible surfactant delivery vehicles, which contain bile salts to improve oral and skin delivery of drugs at various doses [11,12]. Due to the limitations of liposomes and niosomes within the GIT, such as low stability and loading or drug leakage, the development of bilosomes seems promising as an alternative for the same purpose (Figure 1 and Figure 2). 

Additionally, bile salts increase the bioavailability of the drugs via adsorption or permeation into epithelial barriers [13]. Bilosomes improve drug delivery and vaccination against various GIT infectious agents, such as cholera toxin and diphtheria toxins [14,15,16,17]. Due to the intestinal stability and plasticity, bilosomes act as suitable carrier for vaccines and bioactive compounds, as well as antimicrobial, anticancer, and antifungal drugs [16,17,18]. They also find application as alternative drug delivery systems in several other routes, such as ocular and skin treatments [19,20]. Several studies have assessed the antimicrobial potential of bilosomes by carriage of antibiotics or natural bioactive compounds, or vaccines. *Burkholderia pseudomallei*-infected mice were successfully treated with bilosome-loaded levofloxacin and doxycycline, without any deleterious effects on the microbiome. Moreover, levofloxacin-bilosome significantly increased the bactericidal effects of the antibiotic [21]. The luteolin (LL)-loaded pegylated bilosomes (PG-BLs) exhibited higher antibacterial, antioxidant, and anticancer effects than those of single luteolin [22]. Moreover, surface-modified bilosomes synthesized through the solvent evaporation method and loaded with quercetin exhibited higher antibacterial, antioxidant, and anticancer effects than free-quercetin delivery. This system was more efficient against *Escherichia coli* than *Staphylococcus aureus* [23]. In addition, moxifloxacin-loaded bilosome, namely MX-BSop in situ gel (MX-BSop-Ig4), exhibited higher permeation than single moxifloxacin, with advanced antibacterial effects and low tissue toxicity [24]. It also had two- and four-fold lower minimum inhibitory concentrations against *E. coli* and *S. aureus*, respectively. Other compounds, such as quercetin, lycopene, and apigenin, and drugs have been developed for their antibacterial, antiviral, and antifungal effects [25,26,27]. The quercetin-loaded, surface-modified bilosome had a greater antibacterial effect against *E. coli* than *S. aureus* [23]. The antimicrobial potential of bilosome formulations resulted significantly higher than non-formulated pure drugs/compounds and showed less side effects. These lipid carriers have also been applied as vaccine carriers in the forms of liposomes and nano-liposomes against bacterial agents such as group A *Streptococci*, *Helicobacter pylori*, *Yersinia pestis*, and tetanus toxoid, in which the IgA and IgG antibodies were increased. Regarding antiviral effects, or bilosomes containing acyclovir and immunization as vaccine antigen carriers, successful examples come from formulations against Hepatitis B virus (cholera toxin B subunit-conjugated or mannosylated bilosomes), influenza A (H3N2 antigen), and Human enterovirus 71 (HEV71) [28,29,30]. The butenafine (BN)-loaded bilosomes have been developed and demonstrated a significant enhancement in the antifungal activity against *Candida albicans* and *Aspergillus niger*. In this review, the therapeutic and preventive potential of bilosome platforms against the GIT infections were assessed.

## 2. Gastrointestinal Tract Infections

The GIT infections are commonly caused by microorganisms such as viruses, bacteria, fungi, and parasites entering the body through contaminated food or water, contact with infected individuals or animals, or poor hygiene [1,2]. Symptoms of GIT infections can vary depending on the infectious agent, commonly including diarrhea, abdominal pain, nausea, vomiting, fever, and dehydration. Treatment typically involves managing symptoms and addressing the underlying infection through medication, hydration, and rest. Nevertheless, hospitalization may be necessary for severe infections, particularly in young children, older adults, or individuals with suppressed immune systems. Bacterial GIT infections can be caused by several infectious agents, such as *Salmonella*, *Escherichia coli* (*E. coli*), *Campylobacter* spp., *Shigella* spp., and *Clostridium difficile* [1,2]. 

In addition, the most common viruses causing GIT infections include norovirus, rotavirus, and astrovirus. Symptoms of viral GIT infections can vary depending on the viral agent and the severity of infection, but often include diarrhea, vomiting, nausea, abdominal cramps, fever, and dehydration. In more severe cases, symptoms may also include appetite loss, fatigue, headache, and muscle aches. Treatment typically involves managing symptoms, staying hydrated, and allowing the body time to fight off the viral infection. Prevention strategies for viral GIT infections include practicing sufficient hygiene and avoiding close contact with patients, avoiding sharing utensils or drinks with others, and disinfecting contaminated surfaces. Additionally, getting vaccinated against certain types of viral infections, such as rotavirus, can help reduce the risk of contracting a viral GIT infection [31,32]. Fungal GIT infections are caused by various types of fungi, through contaminated food, water, or soil, contact with infected individuals or animals, or poor hygiene. The most common fungal agents of the GIT include *Candida* spp., *Aspergillus* spp., and *Cryptococcus* spp. [33,34]. Parasitic GIT infections are caused by various parasitic agents, such as *Giardia lamblia*, *Cryptosporidium parvum*, *Entamoeba histolytica*, and several species of nematodes, cestodes, and flukes [35,36]. Symptoms of parasitic gastrointestinal infections can vary depending on the type of parasite and the severity of infection, but often include diarrhea, abdominal pain, nausea, vomiting, fever, and dehydration. In more severe cases, symptoms may also include bloody stools, weight loss, and anemia. Treatment typically involves managing symptoms and addressing the underlying parasitic infection through medications, if necessary. Prevention strategies for parasitic GIT infections include practicing good hygiene, avoiding consumption/drinking of contaminated food or water, and avoiding close contact with animals or infected patients. Additionally, proper food storage and preparation, drinking clean water, and avoiding swimming or bathing in potentially contaminated water can also help reduce the risk of parasitic gastrointestinal infections [37,38,39].

## 3. Challenges in the Treatment of Gastrointestinal Infections

There are some challenges in the treatment or drug delivery through the GIT. The development of drug resistance by agents and the low aqueous solubility or toxic effects of drugs has led to developing efficient delivery systems in the complex environment of the GIT [5,6,40,41]. On the other hand, the stability and absorption of drugs through the GIT epithelial cells is often quite poor, leading to the need for proper formulations. Lipid-based nanocarriers offer an alternative with a higher stability and delivery efficiency. For instance, liposomes and niosomes are not sufficiently stable in the GIT considering bile acids and enzymes. Additionally, exosomes have a low capacity for loading drugs. Hence, the recent development and application of bilosomes has yielded acceptable results as these structures contain bile salts and withstand the harsh GIT conditions. Therefore, the efficient formulation of drugs can be optimized to improve their stability, bioavailability, and solubility. Overall, bilosomes offer several advantages as delivery systems for oral drug delivery, including improved stability, increased bioavailability, and controlled release (Table 1). Ongoing research is focused on optimizing these systems for maximizing efficacy and safety [4,42]. Noticeably, the digestibility, oral bioavailability, and local toxicity and side effects of orally prescribed drugs need to be considered when choosing and developing the proper nanocarrier [4,42]. The ability to encapsulate both the hydrophilic and hydrophobic drugs into the same nanocarrier is a great advantage to take into consideration [43]. 

## 4. Lipid-Based Nanocarriers for Oral Drug/Vaccine Delivery 

Lipid-based nanocarriers (bilosomes, niosomes, liposomes, nanocapsules, and nanoemulsions) increase the oral bioavailability of drugs and vaccines, while decreasing the toxicity and side effects [44,45]. Enough solubility, stability, and penetrability to the GIT makes these nanocarriers remarkable delivery systems [46]. These platforms should withstand the adverse GIT environment conditions, which include acid pH, salts, and enzymes. However, not all these systems function efficiently in the wide pH gradient of the GIT to protect the gastric labile drugs. The intricate GIT environment affects the functionality of nanocarriers, as reviewed by Oliver et al. [47]. The intricate human environment has challenges which cannot be overcome using PEG; however, zwitterionic materials, including cationic and anionic groups, reach total neutrality with stronger hydration required for more efficient antifouling traits [48]. The GIT delivery improvement is possible using mucolytic excipients, muco-adhesive nanocarriers (combined with chitosan) [49,50], muco-diffusive nanocarriers (combined with hydrophilic neutral polymers or PEGylated/zwitterionic surfaces/polyglycerol surfactants or protein corona-coating) [51,52,53,54], polysorbate 80 or orlistat corporation, hydrophilic/lipophilic balance, and reduced ester substructures (or use of PEG-ether instead of PEG-ester surfactants) [55,56,57,58]. Chitosan also opens intercellular spaces of enterocytes and facilitates drug penetration. The P-glycoprotein (Pgp) inhibition reduced the drug efflux, while enhancing their adsorption and penetration [59,60]. Phospholipid-containing liposomes (liquid) are disrupted upon exposure to 10 mM bile salts, losing the entire payload [61]. One promising modification to the niosomes included the addition of bile salts and development of bilosomes, increasing paracellular and lymphatic drug uptake [13,18]. Moreover, the addition of fatty acids to the structure is another approach for the GIT delivery optimization [12,62]. Lipid-based nanocarriers function via the opening of tight junctions (SEDDS, micelle, and SLN), endocytosis (micelle and SLN), and transcellular (micelle) and paracellular (SEDDS) penetration [63,64,65,66,67,68,69,70]. The hydrophobic counter-ions commonly include sodium oleate, sodium docusate, sodium dodecyl sulfate, sodium deoxy cholate, sodium taurocholate, tetraheptylammonium bromide, sodium n-octadecyl sulfate, soybean phospholipids, hexadecyl phosphate, sodium n-octadecyl sulphate, and sodium decanoate. 

## 5. Bilosomes and Their Comparison with Liposomes and Niosomes

Bilosomes are major delivery vehicles which prevent vaccines and drugs from degradation in the stomach. As a result, oral delivery of various compounds is possible as an alternative to parenteral therapy. In 2004, nonionic surfactant liposomal structures were synthesized and stabilized using bile salts for oral vaccine delivery [71]. Bilosomes are different from liposomes and niosomes in terms of the composition, storage conditions, and chemical stability (Table 2). Bilosomes were expanded to maintain the antigens’ structure and enhance the mucosal permeability within the GIT. The bilosome-based vaccine has induced a systemic and mucosal immune response similar to that of the subcutaneous injection.

These nonionic surfactant vesicles, in addition to containing bile salts, demonstrate a novel vesicular carrier that acts as an assistant, eliciting considerable immune responses. Bile salts stabilize and protect the bilosomes and their contents from the harsh intestinal environment and enable oral delivery of the vaccine (Figure 2) [72,73].

## 6. Benefits of Bilosomes in Drug and Vaccine Delivery

Bilosomes are stable lipid bilayer structures that can withstand the GIT conditions. Low drug or vaccine leakage compared to liposomes and niosomes, and a higher loading capacity and delivery through the GIT, makes bilosomes promising carriers [11,14,22,42]. Even at low amounts of bilosome-loaded vaccines or antigens, immune cell reactions have occurred. Bilosomes increase the solubility, bioavailability, and rapidity of vaccine and drug delivery, increasing the drug activity and local release, while mitigating their toxicity and side effects. Additionally, bilosomes do not exert any tissue damage and restrain any possible drug side effects [3,12,13,18,21]. Bilosomal formulation of herbal medicines’ bioactive compounds significantly enhanced their antimicrobial and anticancer effects. Several modifications to bilosomes, such as mannosylation and chitosan coating, also enhance their stability and binding to the GIT surface. 

## 7. Disadvantages of Bilosomes 

Despite the promising effects of bilosomes in vaccine and drug delivery through the GIT, consideration of their disadvantages should also be undertaken. For instance, possible high levels of mucosal or systemic immune responses following bilosomal delivery (nano-bilosomes) of antigens/vaccines is a drawback [74]. The current high costs of bilosomes’ synthesis also needs further considerations in order to scale their application in the pharmaceutical industry [75]. 

## 8. Bilosomes Development Techniques

Bilosomes are generally created using the thin-film hydration method [74,75] or the hot homogenization method [29,30,72]. 

### 8.1. Thin-Film Hydration Method

To prepare antigen-containing bilosomes using a surfactant, thin-film hydration, lipid components, cholesterol, and diacetyl phosphate (DCP) are solved and evaporated at a low pressure. The thin layer formed in this way is then hydrated with a buffer including bile salt and antigen to create large multilamellar vesicles, which are transformed into small unilamellar vesicles by extrusion [75]. Thin-layer hydration is utilized to create bilosomes loaded with diphtheria toxoid [75], Hepatitis B antigen [28,74], BSA [76], and tetanus toxoid [73,77].

### 8.2. Hot Homogenization Method

In the hot homogenization process, bilosomes are prepared through melting of the lipid components (cholesterol, monopalmitoyl glycerol, and DCP) at 1408 °C for 5 min, and afterwards, they are hydrated using a buffer solution. After the homogenization of the lipid mixture, bile salt solution is added to form a dispersion including empty vesicles, and then homogenized again. Next, antigen buffer solution is combined with the homogenate and protein entrapment is attained by constant freeze–thaw cycles [78]. 

## 9. Bilosome-Loaded Antibacterial Agents 

Bilosomes have been developed to carry various chemical and natural antibacterial agents. In a study by D’Elia, the in vivo assessment of bilosome-entrapped antibiotics’ (EE, or entrapment efficacy, of 58.9% for levofloxacin and 53.5% for doxycycline) effects against *B. pseudomallei*, the causative agent of melioidosis, was promising in terms of body weight loss, probiotic content, and increased antibacterial activity. Accordingly, levofloxacin had higher detrimental effects on gut microbiota compared to doxycycline in bilosome-free forms, while neither of them exerted anti-microbiome effects in bilosome-formulated platforms. The minimum inhibitory concentrations (MICs) of the free forms of levofloxacin and doxycycline are 2 and 1 µg/mL, respectively. Their minimum bactericidal concentrations (MBCs) are 32 and 16 µg/mL for the free forms of levofloxacin and doxycycline, respectively. The MIC values of bilosome forms of levofloxacin and doxycycline were 4 and 1 µg/mL, respectively. Additionally, their MBC values were 8 and 16 µg/mL, respectively [21].

In a study by Zafar et al., luteolin-loaded PEGylated bilosomes (PG-BLs; 89.52% EE) were developed as an oral delivery route for the antibacterial (against *S. aureus* and *E. coli*) and anticancer/antioxidant assessments. The results revealed a significantly higher activity of the bilosome formulation compared to the free luteolin form. The zone of inhibition (ZOI) of pure ciprofloxacin, luteolin plus PG-BLs, and pure luteolin against *S. aureus* was 17.85 ± 0.15 mm, 16.25 ± 0.13 mm, and 8.5 ± 0.14 mm, respectively. These ZOIs against *E. coli* were, respectively, 18.45 ± 0.11 mm, 14.67 ± 0.14 mm, and 7.23 ± 0.11 mm [23]. 

In another study, surface-modified bilosomes containing quercetin were formulated and their antibacterial and anticancer effects were remarkably higher compared to the quercetin single form. Their effects were seen against MCF-7, t MDA-MB-231, *E. coli*, and *S. aureus* (5 × 10^6^ CFU/mL). The quercetin ZOI against *E. coli* and *S. aureus* was 10.02 ± 0.50 mm and 7.84 ± 0.56 mm, respectively, after 24 h, and 11.66 ± 0.42 mm and 9.15 ± 0.75 mm after 48 h. The bilosome-formulated quercetin (CS-QT-BS3opt1) ZOI against *S. aureus* and *E. coli* was 14.65 ± 0.45 mm and 17.25 ± 0.50 mm, respectively. After 48 h, the CS-QT-BS3opt1 ZOI against *E. coli* and *S. aureus* was 20.76 ± 0.42 mm and 17.54 ± 0.48 mm, respectively [22].

Bilosomes containing moxifloxacin were prepared for ocular delivery and the treatment of *S. aureus* and *E. coli* infection. The alginate containing sol gel exhibited ZOI values of 26.5 ± 1.7 mm and 22.1 ± 1.3 mm against *S. aureus* and *E. coli*, respectively. Additionally a two-fold (0.8 µg/mL vs. 0.4 µg/mL) and four-fold (0.8 µg/mL vs. 0.2 µg/mL) decrease in the MIC was observed against *E. coli* and *S. aureus*, respectively [23]. 

The ciprofloxacin-loaded bilosome (CIP-BLO-opt-IG3) exhibited an EE of 90.14 ± 1.24%, with significantly higher permeability, bio-adhesion property, and antimicrobial activity than pure ciprofloxacin against *Pseudomonas aeruginosa* and *S. aureus*. The pure ciprofloxacin ZOI was 15.81 ± 1.45 mm against *P. aeruginosa* after 24 h. Furthermore, the CIP-BLO-opt-IG3 ZOI was 35.25 ± 1.39 mm against *P. aeruginosa*. The ZOI values of the ciprofloxacin and CIP-BLO-opt-IG3 were 14.42 ± 1.83 mm and 32.74 ± 1.71 mm against *S. aureus* [18]. 

Lycopene isolated from *Lycopersicon esculentum* L. was formulated in bilosomes with an EE of 93.2 ± 0.6% for the oral delivery and antibacterial effect against 32 isolates of *Klebsiella pneumoniae*. Indeed, the increase of bile salts in the bilosome construct resulted in enlargement of vesicles, with an elevated polydispersity index (PDI), a negative charge of zeta potential (ZP), and a decrease in the MIC values, without a significant difference between niosome (F1)- and bilosome (F2)-loaded compounds. Hence, the oral delivery of bilosome formulations in mice decreased the pulmonary fibrosis and enhanced the infection eradication [24]. 

In another study, apigenin (APG)-loaded bilosomes were prepared, and the chitosan coating increased the vesicle size (298 ± 3.56 nm) and enhanced the ZP (+17 mV), muco-adhesion, permeation, and drug release efficacy. The apigenin ZOI was 18 mm against *P. aeruginosa*, *E. coli*, and *Candida albicans*, 20 mm against *Bacillus subtilis*, and 21 mm against *S. aureus* [3]. The encapsulated apigenin in the chitosan-coated bilosome significantly increased the antibacterial activity to 28 mm against *P. aeruginosa*, *B. subtilis*, and *E. coli*, 24 mm against *S. aureus*, and 20 mm against *C. albicans* strains. 

A further study was implemented to assess the stability and potential of bilosomes modified with glucomannan (GM-bilosomes) in provoking the body’s immune response after oral consumption. GM-bilosomes showed favorable qualitative characteristics that simultaneously maintained the chemical and structural stability of tetanus toxin (TT) entrapped in freeze-dried formulations. GM bilosomes showed high stability in various simulated biological fluids and amplified the release profile up to 24 h. Oral GM-bilosomes significantly (*p* < 0.05) induced the systemic immune response compared to the administered free bilosomes, niosomes, and alum-absorbed tetanus toxin. GM-bilosomes were able to induce a mucosal immune response, i.e., sIgA titer in salivary and intestinal secretions, as well as a cellular immune response (IL-2 and IFN-γ levels in spleen homogenate). Accordingly, GM-bilosomes were remarked as a promising carrier and a paramount platform for oral mucosa immunization [77].

## 10. Bilosome-Loaded Antifungal and Antiparasitic Agents 

Species of *Candida*, *Aspergillus*, and *Cryptococcus* cause most fungal gastrointestinal infections. These genera colonize in small numbers in the human body, and their overgrowth following underlying diseases, infections, and suppressed immune systems causes severe diseases [79,80]. Hence, invasive fungal infections are more difficult to eliminate, as compelling evidence has unraveled the potential of opportunistic fungi [34,35,81]. Zafar Ameeduzzafar et al. [28] designed and prepared bilosome-loaded butenafine (BN) using the thin-layer hydration approach. Bilosomes were used to trap water-insoluble compounds to increase penetration across the skin. The prepared optimized gel (BN-BS-og) was further appraised in terms of gel properties, drug release, drug penetration, stimulation, and antifungal studies. The optimized bilosomes displayed an average vesicle size of 215 ± 6.5 nm and an entrapment yield of 89.2 ± 1.5%. BN was completely encapsulated in the lipid matrix of BS. A notably (*p* < 0.05) high release rate (81.09 ± 4.01%) was attained from the optimized bilosomes compared to the prepared optimized gel (65.85 ± 4.87%) and pure butenafine (17.54 ± 1.37%). The penetration rates of BN-BSo, BN-BSog, and pure BN were 56.2 ± 2.7%, 39.2 ± 2.9%, and 16.6 ± 2.3%, respectively. The penetration flux increase ratio for BN-BS-og dispersion and pure BN was 1.4 times and 3.4 times, respectively. This study revealed that BN-BSog was non-allergenic as the score was within the specified range. The antifungal BS-loaded drug activity was significantly increased (*p* < 0.05) against *C. albicans* and *A. niger*. Accordingly, the BS is a substantial platform for transdermal BN delivery. Mannosylated bilosomes could induce significantly higher immune responses compared to the uncoated bilosomes, owing to their higher GIT stability. The O-palmitoylmannan coating increased their affinity to the Peyer’s patches and M-cells, protecting against *Leishmania donovani*. In another study, a combination of methylene blue and curcumin-loaded bilosomes increased the antibacterial and antifungal effects (99.994% and 99.669% mortality, respectively) in a skin infection model [44,45,78]. 

## 11. Bilosome-Loaded Antiviral Agents 

Viral gastroenteritis is a major challenge, particularly among pediatrics < 5years, with considerable mortality requiring timely treatment [32,33,82]. Saifi Zoya et al. [29] investigated an acyclovir-loaded bilosome formulation created using the thin-film hydration method and appraised it for the significant quality properties, such as minimum PDI, optimum particle size, higher medicine entrapment, enhanced dissolution speed, and increased bioavailability. The average vesicle size, dispersion index, and entrapment yield of the optimal formulation of bilosomes were 121.2 ± 3.21 nm, 0.261 ± 0.023, and 83.32 ± 5.46%, respectively. In vitro liberation of bilosome-loaded acyclovir (ACV) was significantly higher (95.1 ± 7.27%) than that of the acyclovir suspension and the supplied formula, 40.23 ± 5.32% and 52.74 ± 5.84%, respectively (at pH 6.8). An ex vivo intestinal penetration study showed a good increase in the influence of bilosomes compared to the ACV solution and the supplied formulation, which was confirmed using confocal laser scanning microscopy, indicating bilosome vesicles’ stability in the GIT. The acokinetic research in Wistar rats exhibited a 4.36- and 2.5-times enhancement in relative bioavailability using bilosomes (*p* < 0.05) at a dose of 5 mg per kg of body weight compared to the ACV suspension and its supplied formulation. Tissue sections of the kidney, liver, and intestine treated with bilosomes showed normal histology at the tested dose for 24 h. Thus, it was deduced that bilosomes are a secure carrier to improve the absorption and bioavailability of the ACV. 

Premanand Balraj et al. [31] investigated the influence of a VP1 (Bac-VP1)-expressing recombinant oral Baculovirus vaccine against Hepatitis E virus (HEV) in a mouse model. The GIT delivery of Bac-VP1 importantly provoked specific IgA and IgG anti-VP1 responses. In addition, the effectiveness of Bac-VP1 associated with bilosomes unveiled that bilosomes loading Bac-VP1 significantly provoked higher responses compared to empty bilosomes. Mice subcutaneous immunization with live Bac-VP1 had remarkably higher VP1-specific serum antibodies compared to oral subjection, while bilosomes enhanced the immunization level, playing a role as an adjuvant against EV71 infections. 

In another study, a wide range of delivery systems, including bilosomes, were considered. Bilayer vesicles made of nonionic surfactants combined with bile salts stabilized the vesicles in the GIT via inhibition of membrane destabilization. The purpose of this research was to probe the efficacy of formulation parameters on bilosome carriers using design experiments to select the appropriate formulation for in vivo evaluation. Bilosomes were made from monopalmitoyl glycerol, cholesterol, diacetyl phosphate, and sodium deoxycholate in different proportions. The optimal formulation of bilosomes was determined and the potential of this formulation as an oral vaccine delivery system was evaluated in biodistribution and vaccine effect studies. Larger sized bilosome vesicles (~6 μm vs. 2 μm diameter) enhanced the uptake in Peyer’s patches and were able to decrease the mean temperature difference change and decrease the viral cell load in an influenza challenge study [30].

Arora, D.A.I.S.Y. et al. [30] demonstrated that antigen-containing mannosylated bilosomes induced a strong immune response against Hepatitis B using a non-invasive administration method. The formulation has revealed remarkably higher resistance than simple antigen and niosomes. The immune response was also appraised, being significantly higher. Elevated levels of antibody (sIgA) were observed at mucosal sites compared to empty bilosomes, while the injected vaccine failed to elicit a significant cellular response. Therefore, broad humoral, cellular, and mucosal immune responses were provoked using the novel, non-invasive vaccine, which could provide long-term protection against the disease.

Another study aimed to boost the findings of bilosomes as a potential oral delivery vehicle for recombinant Hepatitis B surface antigen (HBsAg). This research involved bilosome-loading of the cholera toxin B subunit (CTB) to enhance mucosal absorption via the M-cell-specific delivery approach. The biological activity of CTB, after conjugation, was confirmed by the hemagglutination test. The outcomes displayed that CTB1 generated an anti-HBsAg IgG antibody titer response that was similar to that of the intramuscular (IM) injection of 10 µg of alum-adsorbed HBsAg. In addition, all bilosomal preparations elicited measurable sIgA versus a negligible response with the IM HBsAg injection. Therefore, HBsAg-conjugated CTB bilosomes represent a favorable strategy for oral immunization versus Hepatitis B [29]. Table 3 depicts the bilosomal formulation of drugs and compounds or vaccines against bacterial, fungal, parasitic, and viral agents in various conditions, and their effects. 

## 12. Oral Absorption Enhancement Using Bilosomes 

Bilosomes are stable lipid bilayer structures that can withstand the GIT conditions. Low drug or vaccine leakage compared to liposomes and niosomes, and a higher loading capacity and delivery through the GIT, make bilosomes promising carriers. The lower leakage rates from bilosomes are due to the thicker lipid layer and the presence of the surfactant layer, which help to stabilize the bilayer structure. Liposomes, on the other hand, are composed of a single or multiple lipid bilayers, enclosing an aqueous compartment, and are known to be prone to drug leakage due to their relatively thin lipid layer. Niosomes, which are nonionic surfactant vesicles, also have a tendency for drug leakage due to the formation of defects in the bilayer structure. Even at low amounts of bilosome-loaded vaccines or antigens, immune cells’ provocation has occurred [17,83,84,85,86].

## 13. Future Prospects 

Lipid-based drug or vaccine delivery can be improved or optimized based on the knowledge of the appreciated pros and cons. How to overcome GIT barriers, adsorption, penetration, and the lymphatic and blood circulation of the materials of interest should be considered. This is promising using already known agents or excipients of the structures. The change of the negative to positive charge of bilosomes contributes to the higher cellular uptake of drugs and compounds. The addition of phosphate substructures, polyphosphate, and alkaline phosphatase leads to a positive zeta potential, enhancing GIT adsorption or cellular uptake. Bilosomes can carry large drugs and mask positive charges, making the GIT delivery feasible. These platforms can also systematically distribute drugs. Providing the oily phase, the protein is protected from degradation by enzymes [3,4,5,6,42,74,75]. 

## 14. Conclusions

GIT infections pose an incredible health and economic burden worldwide. The complex nature of the GIT may affect the efficiency of drug adsorption and penetration into the epithelial cells, and then into the lymphatic and blood vessels. The stability, solubility, and bioavailability of oral drugs/vaccines is currently a great concern. Hence, their proper formulation substantially enhances their delivery. Oral delivery also benefits from patients’ compliance and comfortability. Bilosomes are stable lipid bilayer structures that can withstand the GIT conditions. Owing to the structural characteristics, bilosomal loading of drugs or compounds considerably improves the delivery in terms of stability, high loading capacity, bioavailability, and low leakage, compared to those of liposomes and niosomes. It is worth mentioning that bilosomes considerably increase the solubility, bioavailability, and solving rapidity of vaccines and drugs, leading to higher target-specific effects and low side effects in terms of damage to human epithelial cells or microbiota. Bilosomal formulation of herbal medicines’ bioactive compounds has also significantly enhanced their antimicrobial and anticancer effects. Several modifications to bilosomes, such as mannosylation and chitosan coating, have potentiated bilosomes for increased adsorption, and facilitated the delivery of loaded compounds in turn. Considering the scarcity of in vivo studies and clinical trials, there is a need to develop bilosomal formulations for clinical applications. 

## Figures and Tables

**Table 1:** Bilosomes’ applications as delivery systems for drugs and bioactive compounds.

Disease	Drug/Compound	Route of Delivery	Vesicle Size (nm)	Zeta Potential (mV)	PDI	Reference
Viral GIT	Acyclovir	Oral	121.2 ± 3.21	--	0.261	[27]
Respiratory infection	Levofloxacin, doxycycline	Oral	2846.0–3329.33	−23.33, −29.667	--	[21]
Infection and cancer	Luteolin	Oral	252.24 ± 3.54	−32	0.24	[22]
Infection and cancer	Quercetin	Oral	143.51	−15.4	0.256	[23]
Eye infection	Moxifloxacin	Ocular	192 ± 4	−23.5	0.28	[24]
Lung infection	Lycopene	Oral	485.8 ± 35.3	−38.3 ± 4	0.552	[25]
--	Apigenin	--	211 ± 2.87–433 ± 1.98	−15 to −29	<0.5	[3]
Eye infection	Ciprofloxacin	Ocular	182.4 ± 9.2	−34,461.51	0.274	[19]

GIT: gastrointestinal tract, PDI: polydispersity index.

**Table 2:** Comparative characteristics of liposomes, niosomes, and bilosomes.

Parameter	Bilosomes	Liposomes	Niosomes
Composition	Nonionic surfactant and bile salt	Natural phospholipids, cholesterol	Nonionic surfactant with cholesterol
Chemical stability	Stable	Phospholipids undergo the oxidative degradation	Stable
GIT stability	Stable	Unstable	Unstable
Antigen dose	Comparatively low	Comparatively high	Comparatively high
Storage and handling conditions	No special conditions required	Special conditions (liquid nitrogen storage)	No special conditions required

**Table 3:** Bilosomal formulation of drugs and compounds against bacterial, fungal, parasitic, and viral agents in various conditions, and their effects.

Infectious Agent	Drug/Compound	Effects	Study Model	Reference
Bacterial infections				
*B. pseudomallei*	levofloxacin and doxycycline	maintaining normal flora content, increased antibacterial activity	in vitro	[20]
*S. aureus* and *E. coli*	luteolin and ciprofloxacin	increase of antibacterial activity	in vitro	[21]
*S. aureus* and *E. coli*	quercetin	increased anticancer and antibacterial effects	in vitro	[22]
*S. aureus* and *E. coli*	moxifloxacin	increased antibacterial effects	in vitro	[23]
*P. aeruginosa* and *S. aureus*	ciprofloxacin	increased antibacterial effects	in vitro	[19]
*K. pneumoniae*	lycopene	decrease of MIC values	in vitro, in vivo	[24]
*P. aeruginosa*, *E. coli*, and *B. subtilis*	apigenin	significant increase of the antibacterial activity	in vitro	[25]
*C. tetani*	tetanus toxin	increased systemic response	in vivo	[16]
Fungal and parasitic infections				
*C. albicans*	apigenin	significant increase of the antibacterial activity	in vitro	[3,81]
*C. albicans* and *A. niger*	butenafine	significant increase of the antifungal activity	in vitro, in vivo	[24]
*L. donovani*	O-palmitoylmannan	epithelial cells’ protection	in vivo	[24]
*C. albicans* and *S. aureus*	methylene blue and curcumin	antibacterial and antifungal effects	in vitro, in vivo	[82]
Viral infections				
-	acyclovir	increase of absorption and bioavailability,	ex vivo, in vivo	[27]
HEV	Baculovirus vaccine	higher immune responses and serum-specific antibodies	in vivo	[30]
Influenza virus	oral proteins	enhanced uptake by Peyer’s patches	in vivo	[72,79]
HBV	surface antigen/HBsAg and cholera toxin B subunit	enhancement of mucosal absorption	in vivo	[28,29]

*B. pseudomallei*: *Burkholderia pseudomallei*, *S. aureus*: *Staphylococcus aureus*, *E. coli*: *Escherichia coli*, *P. aeruginosa*: *Pseudomonas aeruginosa*, *K. pneumonia*: *Klebsiella pneumonia*, *B. subtilis*: *Bacillus subtilis*, *C. tetani*: *Clostridium difficile*, *C. albicans*: *Candida albicans*, *A. niger*: *Aspergillus niger*, *L. donovani*: *Leishmania donovani*, HEV: Hepatitis E virus, HBV: Hepatitis B virus, HBsAg: Hepatitis B antigen, MIC: minimum inhibitory concentration.

## Data Availability

Not applicable.

## Abbreviations

*B. pseudomallei*: *Burkholderia pseudomallei*, *S. aureus*: *Staphylococcus aureus*, *E. coli*: *Escherichia coli*, *P. aeruginosa*: *Pseudomonas aeruginosa*, *K. pneumonia*: *Klebsiella pneumonia*, *B. subtilis*: *Bacillus subtilis*, *C. tetani*: *Clostridium difficile*, *C. albicans*: *Candida albicans*, *A. niger*: *Aspergillus niger*, *L. donovani*: *Leishmania donovani*, HEV: Hepatitis E virus, HBV: Hepatitis B virus, HBsAg: Hepatitis B antigen, MIC: minimum inhibitory concentration, ACV: acyclovir, BN: butenafine, BN-BS-og: the prepared optimized gel, BS: bilosome, CTB: cholera toxin B subunit, DCP: diacetyl phosphate, GM-bilosomes: glucomannan-modified bilosomes, HBsAg: Hepatitis B surface antigen, IM: intramuscular, NISV: nonionic surfactant vesicle, TT: tetanus toxoid.
